# Curvature-Aware Point-Pair Signatures for Robust Unbalanced Point Cloud Registration

**DOI:** 10.3390/s25206267

**Published:** 2025-10-10

**Authors:** Xinhang Hu, Zhao Zeng, Jiwei Deng, Guangshuai Wang, Jiaqi Yang, Siwen Quan

**Affiliations:** 1 School of Electronics and Control Engineering, Chang’an University, Xi’an 710064, China; 2024132040@chd.edu.cn (X.H.); zhaozeng@chd.edu.cn (Z.Z.); 2 China Railway Design Corporation, Tianjin 300251, China; dengjiwei@crdc.com (J.D.); wangguangshuai@crdc.com (G.W.); 3 The School of Computer Science, Northwestern Polytechnical University, Xi’an 710129, China; jqyang@nwpu.edu.cn

**Keywords:** unbalanced point cloud registration, one-to-many correspondences, local point cluster structure

## Abstract

Existing point cloud registration methods can effectively handle large-scale and partially overlapping point cloud pairs. However, registering unbalanced point cloud pairs with significant disparities in spatial extent and point density remains a challenging problem that has received limited research attention. This challenge primarily arises from the difficulty in achieving accurate local registration when the point clouds exhibit substantial scale variations and uneven density distributions. This paper presents a novel registration method for unbalanced point cloud pairs that utilizes the local point cluster structure feature for effective outlier rejection. The fundamental principle underlying our method is that the internal structure of a local cluster comprising a point and its K-nearest neighbors maintains rigidity-preserved invariance across different point clouds. The proposed pipeline operates through four sequential stages. First, keypoints are detected in both the source and target point clouds. Second, local feature descriptors are employed to establish initial one-to-many correspondences, which is a strategy that increases correspondences redundancy to enhance the pool of potential inliers. Third, the proposed Local Point Cluster Structure Feature is applied to filter outliers from the initial correspondences. Finally, the transformation hypothesis is generated and evaluated through the RANSAC method. To validate the efficacy of the proposed method, we construct a carefully designed benchmark named KITTI-UPP (KITTI-Unbalanced Point cloud Pairs) based on the KITTI odometry dataset. We further evaluate our method on the real-world TIESY Dataset which is a LiDAR-scanned dataset collected by the Third Railway Survey and Design Institute Group Co. Extensive experiments demonstrate that our method significantly outperforms the state-of-the-art methods in terms of both registration success rate and computational efficiency on the KITTI-UPP benchmark. Moreover, it achieves competitive results on the real-world TIESY dataset, confirming its applicability and generalizability across diverse real-world scenarios.

## 1. Introduction

Point cloud registration (PCR) is a fundamental task in 3D computer vision [1,2], which aims to estimate a six-degree-of-freedom (6-DoF) rigid transformation so that point clouds can be precisely aligned. This technique plays a critical role in various applications, including autonomous vehicle localization, 3D object detection [3,4], and large-scale scene reconstruction [5]. While existing methods assume scale-balanced inputs, practical scenarios frequently necessitate unbalanced registration, where a local partial scan (e.g., single LiDAR frame) must be aligned with a global environmental map (e.g., city-scale 3D model) [6,7]. This paradigm introduces substantial challenges primarily arising from extreme scale disparities that significantly amplify outlier ratios [8,9,10]. Non-uniform density distributions further suppress discriminative local structures during feature extraction [11,12], while performance degradation becomes particularly pronounced in low-overlap scenarios [12]. The presence of repetitive architectural patterns also contributes to matching ambiguities in large-scale environments [13].

Despite substantial progress in handling low-overlap and high-outlier scenarios [11,14], current point cloud registration methods exhibit critical limitations when faced with unbalanced inputs characterized by significant differences in spatial extent and point density [15,16]. Feature-based methods leveraging matchability detection [11] or hierarchical correspondence prediction [13] frequently succumb to descriptor indistinctiveness as sparse local structures interact with dense environmental regions, while deep learning-based registration networks optimized for balanced inputs demonstrate poor generalization to extreme scale variations [12,17]. Furthermore, global localization frameworks that discretize large-scale maps into fragmented components [18,19] introduce artificial segmentation artifacts, fundamentally compromising holistic alignment integrity. Crucially, all methods suffer from structural distractions that repetitive geometric patterns in expansive environments misguide feature matching [11], compounded by density-induced bias that drowns discriminative local features within high-density zones of global point clouds [12].

To address these challenges, we propose a point-pair signature method that is aware of curvature (CURV) and specifically designed for unbalanced point cloud registration. Our main contributions are summarized as follows:

- Curvature-optimized keypoint detection that significantly outperforms existing methods (e.g., ISS, H3D) [8,20,21,22,23] in repeatability under scale variations.
- Systematic investigation of one-to-N correspondence for unbalanced PCR, demonstrating its superiority over conventional one-to-one matching paradigms [24,25].
- By integrating curvature-aware signatures with geometric consistency validation, our rejection mechanism simultaneously improves inlier selection ratios, maintains registration accuracy, and ensures computational efficiency [9,26,27].

This paper is structured as follows: Section 2 reviews the existing literature on point cloud registration, covering both balanced and unbalanced scenarios. Section 3 details our method, including problem formulation, keypoint detection via voxel downsampling curvature estimation, one-to-many correspondences generation using feature similarity, and local cluster-based matching. Section 4 presents the experimental setup and evaluation results validating our method. Section 5 draws the conclusions and discusses future research directions.

## 2. Related Work

This section provides a brief overview of the existing point cloud registration methods, including balanced and unbalanced registration methods.

### 2.1. Balanced Point Cloud Registration

Recent advances in point cloud registration methodologies can be broadly categorized into two main methods: traditional geometric methods and deep learning-based methods.

For geometric methods, the classical RANSAC framework [8,20,21,25,26,28] remains fundamental for 6-DoF pose estimation. Several improved variants have been developed, including SAC-IA (utilizing spatial uniform sampling) [24], GC-RANSAC (employing graph-cut optimization) [8], and SAC-COT (based on compatibility graph sampling) [25]. Complementary global optimization methods such as GO-ICP [27] and GORE [9] have been proposed, implementing intelligent ICP [29] scheduling and precise boundary computation, respectively.

Deep learning-based methods have introduced various novel architectures for point cloud registration. Some methods, such as FCGF [30] and D3Feat [11], employ fully convolutional designs and joint learning frameworks for feature extraction. Architectures such as Predator [12] utilize attention mechanisms to address low-overlap scenarios, while SpinNet [1] incorporates rotation-invariant designs. Additionally, some methods like PointDSC [11] and RGM [31] apply deep learning and graph matching techniques for inlier/outlier differentiation. Recent detection-free methods [32], particularly CoFiNet [17] and GeoTransformer [33], focus on the transformation parameter estimation by an end-to-end manner.

Despite these advancements, both methodological categories present certain limitations. Traditional geometric methods face computational challenges in high-outlier scenarios [10] and demonstrate sensitivity to point cloud scale variations [9,27]. Deep learning-based methods remain constrained by their dependence on extensive training data and exhibit limited generalization capabilities [12,13,34]. Emerging hybrid methods such as MAC [14,35] attempt to address these limitations by integrating geometric- and learning-based advantages. Future research should focus on developing more efficient and robust registration algorithms capable of handling complex 3D scenarios in practical applications.

### 2.2. Unbalanced Point Cloud Registration

Unbalanced registration addresses scenarios characterized by extreme disparities in spatial scale or point density, exemplified by the alignment of large-scale reconstructed maps with local scans [7,15,36,37]. Existing methods can be broadly categorized into two paradigms.

The first is the localization paradigm, which adopts a two-stage “retrieval + local registration” framework: global descriptors like NetVLAD [7] first retrieve the most relevant reference frame from a prebuilt map, followed by balanced pairwise registration. Although some methods such as Du [16] attempt joint optimization of local and global features, this framework suffers from fundamental limitations [38]. Significant computational and memory overhead arises from storing numerous overlapping reference frames [18,19], leading to memory explosion. The method also fails to handle domain differences; for instance, severe density gaps between sparse SfM point clouds and dense LiDAR scans cause feature matching to collapse [39]. Crucially, errors from the retrieval stage propagate irreversibly—if coarse alignment fails, subsequent registration cannot recover [38].

The second is the registration paradigm, where existing robust estimators catastrophically fail under unbalanced conditions. RANSAC assumes uniform point distributions [8,20], causing ineffective minimal set sampling in density-variant regions. BnB methods [9,10,27] rely on spatial consistency for bound computation, but scale discrepancies induce overly loose bounds that nullify optimality guarantees. Spatial compatibility-based outlier filters [35] misclassify correspondences in non-overlapping areas due to geometric asymmetry. Although specialized pipelines such as Lu [11] target niche challenges (e.g., large-scale LiDAR registration), no unified framework addresses unbalanced registration holistically [13,40]. The core conflict lies in the inherent “equilibrium assumption” of traditional methods clashing with the geometric heterogeneity of unbalanced scenarios, demanding fundamentally adaptive mechanisms for scale and density variations.

## 3. Method

**Figure 1:**
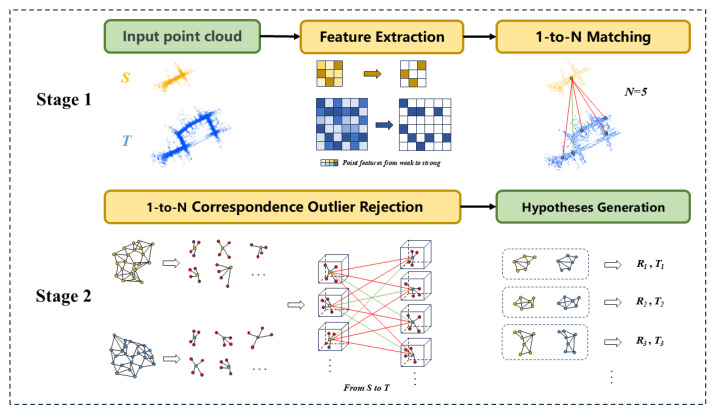
Pipeline of the proposed method. **(1)** Source point cloud and target point cloud. **(2)** Keypoint Detection. **(3)** One-to-N Correspondence Matching (*N* = 5). **(4)** Each point and its K-nearest neighbors (e.g., k = 4) form a local geometric structure for similarity matching. **(5)** Hypothesis Generation.

Our method is illustrated in Figure 1, registering a partial-scanned source point cloud to a large-scale target under a significant scale difference. First, keypoints are extracted from two point clouds based on curvature information. Second, initially dense correspondences are generated via the FPFH [24] descriptor and feature matching method for both point clouds. Finally, a novel point-pair signature is introduced to remove outliers from the initial correspondences, and then the 6-DoF transformation is estimated through robust hypothesis evaluation.

### 3.1. Problem Formulation

Given source point cloud P and target point cloud Q, which are downsampled from the original partial-view source point cloud $S\in R^{n\times 3}$ and the complete target point cloud $T\in R^{m\times 3}$, respectively, with n≪m indicating a substantial scale difference. A correspondence pair C=(p,q) represents a feature matching from a point *p* in P and a point *q* in Q. The goal of registration is to find a 6-DoF rigid transformation, composed of a rotation matrix *R* and a translation vector *t*, that optimally aligns P to *Q* such that q=R·p+t holds for corresponding points.

### 3.2. Keypoint Detection

The critical innovations introduced by our curvature-aware detection module are fundamentally different from the traditional keypoint detectors like ISS [22]. While ISS [22] typically employs fixed threshold and uniform neighborhood processing, our method utilizes a normalized eigenvalue ratio $\kappa_{i}^{p}=\frac{\lambda_{0}}{\lambda_{0}+\lambda_{1}+\lambda_{2}+\epsilon }$ that prioritizes subtle geometric features through the smallest eigenvalue, significantly enhancing sensitivity to scale variations. Furthermore, our integrated pipeline combines adaptive threshold $\tau_{\kappa }$ with hysteresis-based NMS using a dynamic radius $r_{n}=\alpha r_{v}$ (α∈[2,5]), ensuring robust keypoint selection across non-uniform point densities and reducing noise sensitivity through second-order differential properties. The unique combination of density invariance, noise robustness, and geometric discriminability specifically addresses the inherent challenges of unbalanced scenarios, including extreme scale disparities and heterogeneous density distributions.

The practical implementation of our strategy relies on the strategic configuration of two core parameters: the voxel size $r_{v}$ and curvature threshold $\tau_{\kappa }$. The voxel size $r_{v}$ functions as a physical-scale normalizer, with its selection guided by the characteristic scale of salient geometric features in the target environment, providing inherent adaptability to different scene scales and point densities. Complementarily, the curvature threshold $\tau_{\kappa }$ governs the essential trade-off between keypoint repeatability and distinctiveness, configurable through analysis of curvature value distributions in representative data samples. The principled yet flexible parameter selection strategy underpins the method’s consistent performance across diverse datasets and sensing conditions, as evidenced by our results on both KITTI-UPP and TIESY benchmarks, representing a key strength for handling the inherent variations in unbalanced point cloud registration.

Given source point cloud P and target point cloud Q, we perform keypoint detection through curvature analysis as follows:

#### 3.2.1. Voxel Downsampling

Both point clouds undergo voxel grid filtering for computational efficiency:

(1) $$\begin{array}{cc} P^{\prime } & =\mathrm{VoxelDownSample}(P,r_{v}), \end{array}$$

(2) $$\begin{array}{cc} Q^{\prime } & =\mathrm{VoxelDownSample}(Q,r_{v}), \end{array}$$

where $r_{v}$ is the voxel edge length controlling the downsampling resolution. This operation preserves geometric features larger than $r_{v}$ while reducing point density.

#### 3.2.2. Local Curvature Estimation

For each point $p_{i}\in P^{\prime }$ and $q_{j}\in Q^{\prime }$, compute the covariance matrix over its $r_{c}$-radius neighborhood:

(3) $$\begin{array}{cc} {𝒩}_{i}^{p} & =\{p_{j}|∥p_{j}-p_{i}{∥}_{2}\leq r_{c}\}, \end{array}$$

(4) $$\begin{array}{cc} C_{i}^{p} & =\frac{1}{|{𝒩}_{i}^{p}|}\sum_{p_{j}\in {𝒩}_{i}^{p}}\left(p_{j}-{\bar{p}}_{i}\right){\left(p_{j}-{\bar{p}}_{i}\right)}^{T}. \end{array}$$

Through the eigen decomposition $C_{i}^{p}=V\Lambda V^{T}$ ($\Lambda =\mathrm{diag}(\lambda_{0},\lambda_{1},\lambda_{2})$), the curvature is as follows:

(5) $$\kappa_{i}^{p}=\frac{\lambda_{0}}{\lambda_{0}+\lambda_{1}+\lambda_{2}+\epsilon },\;\epsilon =10^{-8},$$

where $\lambda_{0}\leq \lambda_{1}\leq \lambda_{2}$ encode the local surface variation. The same process is applied to $Q^{\prime }$ to obtain $\kappa_{j}^{q}$.

#### 3.2.3. Candidate Keypoint Selection

Select geometrically salient points from both clouds:

(6) $$\begin{array}{cc} K_{p} & =\{p_{i}\in P^{\prime }|\kappa_{i}^{p}>\tau_{\kappa }\}, \end{array}$$

(7) $$\begin{array}{cc} K_{q} & =\{q_{j}\in Q^{\prime }|\kappa_{j}^{q}>\tau_{\kappa }\}, \end{array}$$

where the $\tau_{\kappa }$ is a corresponding threshold set for keypoint selection.

#### 3.2.4. Non-Maximum Suppression

The refined keypoints are selected through spatial competition:

(8) $$\begin{array}{cc} P_{\mathrm{key}} & =\left\{p_{i}\in K_{p}|\kappa_{i}^{p}>\max_{p_{j}\in B\left(p_{i},r_{n}\right)}\left(\kappa_{j}^{p}-\delta \right)\right\}, \end{array}$$

(9) $$\begin{array}{cc} Q_{\mathrm{key}} & =\left\{q_{j}\in K_{q}|\kappa_{j}^{q}>\max_{q_{k}\in B\left(q_{j},r_{n}\right)}\left(\kappa_{k}^{q}-\delta \right)\right\}, \end{array}$$

where $B\left(p_{i},r_{n}\right)=\{p_{j}|∥p_{j}-p_{i}{∥}_{2}\leq r_{n}\}$, $r_{n}=\alpha r_{v}$ (α∈[2,5]), and δ introduce hysteresis.

### 3.3. One-to-Many Correspondances

The proposed one-to-many matching strategy provides an effective solution to the feature asymmetry challenge inherent in unbalanced point cloud registration. Building upon the detected keypoints $P_{\mathrm{key}}$ and $Q_{\mathrm{key}}$ from Section 3.2, our method establishes multiple potential correspondences for each source keypoint, creating a redundant yet discriminative matching space. This design offers two key benefits: (1) the increased correspondence candidates significantly improve inlier counts for final registration; (2) the adaptive feature extraction pipeline maintains geometric consistency while accommodating density variations.

The core innovation of our method lies in its integrated handling of two critical aspects: local feature representation and correspondence validation. For feature representation, we employ FPFH descriptor with adaptive radius scaling to dynamically adjust to non-uniform point distributions. For correspondence validation, the reciprocal $L_{2}$-norm similarity metric provides robust constraints in feature space, effectively suppressing mismatches from non-overlapping regions. This combination ensures both the quantity and quality of matches, addressing the fundamental challenges in unbalanced registration.

#### 3.3.1. Feature Extraction

The FPFH features are computed from source and target keypoints:

(10) $$\begin{array}{cc} F_{s} & =\{\phi \left(p\right)|p\in P_{\mathrm{key}}\}, \end{array}$$

(11) $$\begin{array}{cc} F_{t} & =\{\phi \left(q\right)|q\in Q_{\mathrm{key}}\}, \end{array}$$

where ϕ(·) denotes the FPFH descriptor, computed with adaptive radius search:

(12) $$r=\alpha \cdot d,\;d=\frac{1}{\left|K\right|}\sum_{k=1}^{\left|K\right|}∥p_{k}-{\mathrm{NN}}_{K}\left(p_{k}\right)∥.$$

Here K represents the keypoint set ($P_{\mathrm{key}}$ for source, $Q_{\mathrm{key}}$ for target), *d* is the average spacing between keypoints, and α is a scale factor. The nearest neighbor ${\mathrm{NN}}_{K}\left(p_{k}\right)$ is searched within the same keypoint set.

#### 3.3.2. Similarity Metric

This curvature-aware similarity metric facilitates robust correspondence establishment by quantifying the geometric affinity between locally salient points across different point clouds. The feature similarity is defined using $L_{2}$-norm reciprocal:

(13) $$s\left(f_{i},f_{j}\right)=\frac{1}{1+∥f_{i}-f_{j}{∥}_{2}}.$$

#### 3.3.3. One-to-Many Correspondence Generation

For each source keypoint $p\in P_{\mathrm{key}}$, find the ${\mathrm{top}}_{N}$ target keypoints with the highest similarity:

(14) $$T\left(p\right)=\underset{q\in Q_{\mathrm{key}}}{{\mathrm{top}}_{N}}\left\{s\left(\phi \left(p\right),\phi \left(q\right)\right)\right\},$$

where ${\mathrm{top}}_{N}$ selects the *N* points with maximum similarity. The initial correspondence set is generated as follows:

(15) $$C_{\mathrm{initial}}=\underset{p\in P_{\mathrm{key}}}{⋃}\left\{(p,q)|q\in T(p),s(\phi (p),\phi (q))>\theta \right\}.$$

This strategy generates up to $|P_{\mathrm{key}}|\times N$ keypoint-level correspondences. The redundant design boosts inlier quantity while feature similarity constraints (θ=0.2) filter interference from non-overlapping regions.

### 3.4. Local Geometric Verification

**Figure 2:**
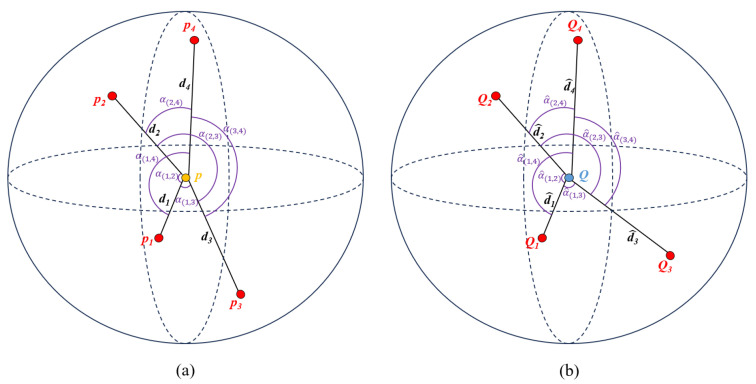
Visualization of the local geometric structure comparison for a candidate correspondence (p,q), shown with k=4 neighbors as a representative example. The source point *p* (**a**) and its *k* nearest neighbors form a local structure characterized by radial distances $d_{i}=\left|p-p_{i}\right|$ (ordered as $d_{1}\leq d_{2}\leq \ldots \leq d_{k}$) and angular relationships $\alpha_{\left(i,j\right)}=∠\left(p_{i}pp_{j}\right)$ between neighbor pairs. Similarly, the target point *q* (**b**) exhibits corresponding features $\hat{d}i=\left|q-q_{i}\right|$ and $\hat{\alpha }\left(i,j\right)=∠\left(q_{i}qq_{j}\right)$. The similarity matching process directly compares these distance and angle features between the two structures to verify correspondence validity.

The local geometric verification module establishes robust correspondences by examining the structural consistency between keypoints and their *k*-nearest neighborhoods (illustrated using =4), as visualized in Figure 2. For each candidate pair $\left(p,q\right)\in C_{\mathrm{initial}}$, ordered neighbor sets $𝒩p=p_{i}$ and $𝒩q=q_{i}$ (sorted by Euclidean distance $|p-p_{i}|$) are extracted to calculate two complementary geometric descriptors: (1) relative distance features $d_{i}=\left|p-p_{i}\right|$ and $\hat{d}i=\left|q-q_{i}\right|$, and (2) angular consistency measures $\alpha_{\left(i,j\right)}$ and ${\hat{\alpha }}_{\left(i,j\right)}$. The binary agreement indicators $M_{d}\left(i\right)$ and $M_{\alpha }\left(i,j\right)$ are then derived by adaptive thresholds $\delta_{d}$ and δα, with the final similarity score S(p,q) calculated as a normalized consensus of matched characteristics.

This dual feature paradigm addresses a critical limitation of conventional methods, which often solely rely on distance metrics, by introducing angular coherence as an orthogonal validation signal. Furthermore, the adaptive thresholding mechanism dynamically adjusts to local point density variations, ensuring reliable performance even under significant noise. In particular, the neighborhood-preserving design guarantees that local structures from sparser point clouds remain identifiable within denser counterparts, overcoming a key challenge in unbalanced registration scenarios.

Building upon the initial correspondences in Section 3.3, geometric verification is performed through local structure matching. Each candidate (p,q) is evaluated by comparing its *k*-NN geometric constellation, achieving computational efficiency via feature evaluation while outperforming global verification methods in outlier rejection. The balance of precision and scalability makes the method particularly suitable for real-time applications where structural fidelity must be preserved without compromising processing speed.

#### 3.4.1. Local Structure Construction

For each keypoint $p\in P_{\mathrm{key}}$ and $q\in Q_{\mathrm{key}}$, its local geometric structure is constructed using its *k* nearest neighbors:

(16) $$\begin{array}{cc} {𝒩}_{p} & =\left\{p_{i}|i=1,2,\ldots ,k\right\}\;∥p-p_{i}∥\leq ∥p-p_{i+1}∥, \end{array}$$

(17) $$\begin{array}{cc} {𝒩}_{q} & =\left\{q_{i}|i=1,2,\ldots ,k\right\}\;∥q-q_{i}∥\leq ∥q-q_{i+1}∥, \end{array}$$

where the neighbors are ordered by the Euclidean distance from the central point.

#### 3.4.2. Local Structure Similarity Matching

The geometric features and their matching criteria are defined as follows:

(18) $$\begin{array}{cc} d_{i} & =∥p-p_{i}∥,\;{\hat{d}}_{i}=∥q-q_{i}∥,\;i\in \left\{1,2,\ldots ,k\right\}, \end{array}$$

(19) $$\begin{array}{cc} \alpha_{\left(i,j\right)} & =∠\left(p_{i}pp_{j}\right),\;{\hat{\alpha }}_{\left(i,j\right)}=∠\left(q_{i}qq_{j}\right),\;1\leq i<j\leq k. \end{array}$$

The $d_{i}$ and ${\hat{d}}_{i}$ are the distance features. The $\alpha_{\left(i,j\right)}$ and ${\hat{\alpha }}_{\left(i,j\right)}$ are the angle features. For each feature, a binary match indicator is defined based on the threshold conditions:

(20) $$\begin{array}{cc} \mathrm{Distance}\;\mathrm{matching}:\;M_{d}\left(i\right) & =\left\{\begin{array}{cc} 1 & \mathrm{if}\;|d_{i}-{\hat{d}}_{i}|<\delta_{d}\\ 0 & \mathrm{otherwise} \end{array}\right., \end{array}$$

(21) $$\begin{array}{cc} \mathrm{Angle}\;\mathrm{matching}:\;M_{\alpha }\left(i,j\right) & =\left\{\begin{array}{cc} 1 & \mathrm{if}\;|\alpha_{\left(i,j\right)}-{\hat{\alpha }}_{\left(i,j\right)}|<\delta_{\alpha }\\ 0 & \mathrm{otherwise} \end{array}\right., \end{array}$$

where the $\delta_{d}$ is a distance difference threshold, and the $\delta_{\alpha }$ is an angle difference threshold.

The similarity score is then computed as the proportion of matched features:

(22) S(p,q)=∑i=1kMd(i)+∑1≤i<j≤kMα(i,j)k+k2,

where the *k* is the number of nearest neighbors and the k2=k(k−1)2.

#### 3.4.3. Matched Point Pairs Generation

Final correspondences are established by selecting candidate pairs that satisfy the similarity threshold condition:

(23) $$M=\left\{\left(p,q\right)|\left(p,q\right)\in C_{\mathrm{initial}},\;S\left(p,q\right)>\tau \right\},$$

where τ is a similarity threshold that controls the matching strictness and ensures correspondence quality.

### 3.5. Hypothesis Generation and Evaluation

Based on the filtered correspondence set M obtained from outlier rejection, the number of outliers in the point correspondence set is significantly reduced, thus we can directly employ the RANSAC algorithm for robust transformation estimation. We employ the RANSAC algorithm to robustly estimate the optimal 6-DoF rigid transformation (R,t). The algorithm iteratively performs the following procedure: in each iteration, three point correspondences are randomly selected to compute a rigid transformation hypothesis (R,t) via SVD, followed by inlier counting with threshold γ (where ${\left|Rp+t-q\right|}_{2}<\gamma$) [41,42,43]. After typically thousands of iterations, the hypothesis with the maximum inliers is selected and refined using its consensus set to obtain the final transformation estimate.

To quantitatively evaluate the registration accuracy, we computed the rotation error and translation error against the ground truth transformation (Rgt,tgt). After multiple rounds of optimization, the best hypothesis is refined to obtain the final transformation. The registration result is evaluated using the following:

(24) $$\begin{array}{cc} \mathrm{RE} & =\arccos\left(\frac{\mathrm{tr}(R^{*}R_{gt}^{T})-1}{2}\right)\times \frac{180}{\pi }, \end{array}$$

(25) $$\begin{array}{cc} \mathrm{TE} & =∥t^{*}-t_{gt}{∥}_{2}. \end{array}$$

The rotation error (RE) is computed using the trace operator tr(·), which sums the diagonal elements of a matrix. Specifically, RE quantifies the angular deviation in degrees between the estimated and ground truth rotations. And the translation error (TE) measures the Euclidean distance between the estimated and ground truth translation vectors. These metrics provide complementary perspectives on registration quality, with RE quantifying rotational alignment and TE quantifying positional accuracy. Lower values for both metrics indicate better registration performance.

## 4. Experiments

### 4.1. Experimental Setup

**Figure 3:**
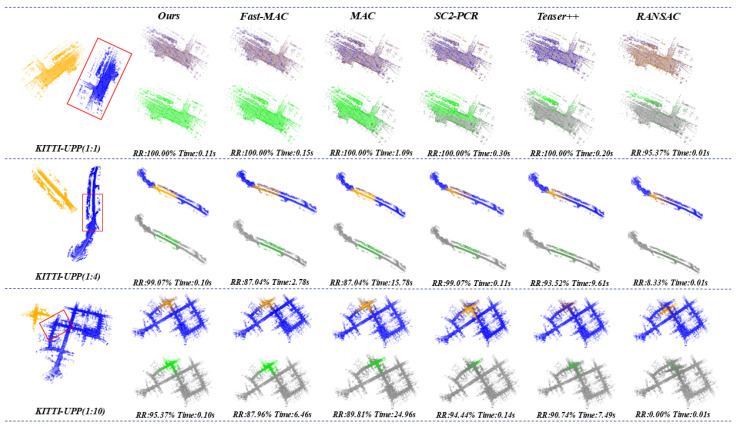
Registration visualizations of various methods on KITTI-UPP dataset. The pre and post registration are shown, with green color depth quantitatively indicating alignment accuracy.

**Figure 4:**
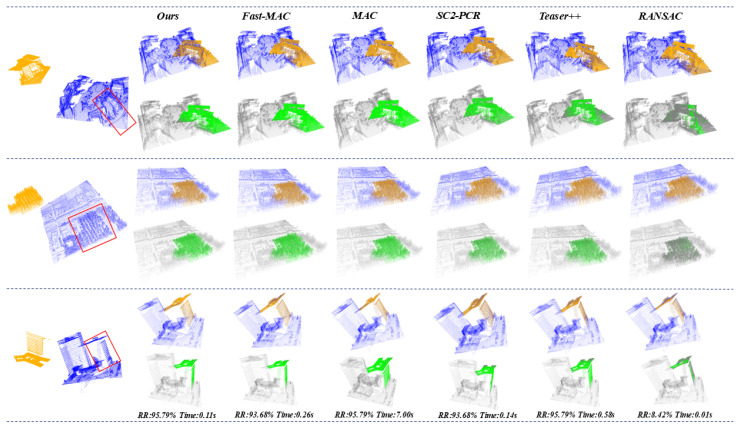
Registration visualizations of various methods on real-world TIESY dataset. The pre and post registration are shown, with green color depth quantitatively indicating alignment accuracy.

Datasets. Our method is evaluated on two complementary datasets:

- Synthetic KITTI-UPP: Created from KITTI Odometry with fixed sampling interval (hop=10) to control unbalance ratios [44]:
  - Balanced (1:1): 150-frame query vs. 150-frame reference
  - Moderate Unbalance (1:4): 150-frame query vs. 600-frame reference
  - Severe Unbalance (1:10): 150-frame query vs. 1500-frame reference
  Each group contains 108 registration pairs with query frames strictly excluded from reference point clouds. The unbalanced ratios (1:1, 1:4, 1:10) precisely define the relative scale between source and target point clouds based on frame aggregation ranges. For example, the 1:10 ratio configuration involves registration between a source point cloud aggregated from frames 0–150 and a target point cloud aggregated from frames 0–1500, ensuring the source is entirely contained within the target. Some samples are visualized in Figure 3.
- Real-world TIESY dataset: Collected via mobile LiDAR scanning in diverse urban/rural environments by TIESY Survey Institute, featuring natural unbalance ratios of 1:4 to 1:6. This dataset comprises 95 registration pairs across 18 geographical areas, covering streets, buildings, vegetation, and infrastructure. Some samples of the dataset are visualized in Figure 4.

Evaluation Criteria. For both the KITTI-UPP dataset and the real-world TIESY dataset, we employ the RE and TE as the evaluation metrics. The registration is considered successful when RE ≤ 15° and TE ≤ 60 cm. A dataset’s registration accuracy of a method is defined as the ratio of successful cases to the total number of point cloud pairs.

Implementation Details. In our experiments which are designed to evaluate performance under varying unbalanced conditions, we adopt appropriate values of *N* (which determines the one-to-many matching pairs) for the three KITTI-UPP ratios: N=1 for the 1:1 ratio, N=12 for the 1:4 ratio, and N=20 for the 1:10 ratio. Furthermore, for the scenario involving large-scale point clouds aggregated by 3000 frames, we maintain the parameter *N* at 20. For the real-world TIESY dataset, all the results we selected are tested with the parameter N set to 12. Regarding the local point cluster structure that we utilize, the number of neighboring points *k* is set to four, while the angle and distance thresholds are configured as 5° and 0.1 m, respectively.

### 4.2. Results on KITTI-UPP Dataset

#### 4.2.1. Evaluation on One-to-Many Correspondences

Building upon our evaluation framework, an evaluation of one-to-many correspondence generation is conducted using ISS [22] keypoints on the KITTI-UPP dataset with 1:4 ratio. The assessment examines two distinct registration scenarios: (1) Small-to-Large: registration from partial scans to the whole; (2) Large-to-Small: registration from the whole scans to the partial.

In the experiments, the parameter *N* is varied across the following discrete values:

*Small-to-Large*: N∈{1,4,6,8,12,16,20,24};

*Large-to-Small*: N∈{1,2,4,6}.

Six methods including Fast-MAC [43], MAC [41], SC2-PCR [42], Teaser++ [45], FGR [46], and RANSAC [28] are rigorously evaluated under both configurations. Comprehensive performance results, including registration rates and error metrics, are provided in Table 1. Cases where Fast-MAC [43], MAC [41], and Teaser++ [45] fail to process due to excessive correspondence pairs or memory overflow are treated as registration failures and reflected in the registration success rate. Specifically, this occurs for MAC and Fast-MAC at *N* = 16, 20, and 24 in the small-to-large scenario and at *N* = 4, and 6 in the large-to-small scenario, while Teaser++ encounters similar limitations at *N* = 24 in the small-to-large scenario and at *N* = 4, and 6 in the large-to-small scenario.

As evidenced by the results in Table 1, our method consistently achieves superior performance under all experimental configurations. (1) A key observation is the significant performance improvement across nearly all methods as *N* increases. For instance, when *N* is increased from 1 to 12, the RR of methods such as Fast-MAC [43], MAC [41], and SC2-PCR [42] significantly improves from 51.85% to 97.22%, strongly validating the effectiveness of the one-to-many correspondence strategy. (2) While Fast-MAC [43], MAC [41], and Teaser++ [45] exhibit improved accuracy with larger *N* values, they often incur substantial computational overhead or even fail to process dense correspondence sets efficiently, resulting in performance degradation. When *N* is increased from 12 to 24, the registration time of methods such as Fast-MAC [43] and MAC [41] increases significantly, while the registration rate drops markedly from 97.22% to 26.85%. In contrast, our method not only attains the highest registration accuracy but also maintains the lowest computational time, demonstrating remarkable efficiency even at N=24. (3) Our method retains competitive performance under large value of *N* when the keypoint detection module is substituted with ISS [22] method.

#### 4.2.2. Experiments with Different Keypoint Modules

We conduct extensive experiments on the KITTI-UPP dataset under three unbalanced registration scenarios at ratios of 1:1, 1:4, and 1:10. Our evaluation framework incorporates multiple keypoint detection methods, including ISS [22], H3D [23], and our curvature-based method. As shown in Table 2, we report comprehensive comparisons with both traditional methods, including Fast-MAC [43], MAC [41], SC2-PCR [42], Teaser++ [45], FGR [46], and RANSAC [28], as well as deep learning-based methods, specifically GeoTransformer [33] and PARENet [47].

The comprehensive comparison demonstrates that our method achieves superior performance when utilizing curvature-based keypoints, particularly in severely unbalanced scenarios. Under the “CURV + FPFH“ configuration, our method not only attains the highest registration success rates across all imbalance ratios (100.00%, 99.07%, and 95.37%, respectively) but also maintains exceptional computational efficiency with consistently low time consumption (0.11 s, 0.10 s, and 0.10 s, respectively). This performance advantage becomes particularly evident in the most challenging 1:10 ratio scenario where our method outperforms all competing methods by achieving the highest registration success rate while simultaneously maintaining significantly superior computational efficiency compared to other high-performance methods. By contrast, deep learning-based methods that GeoTransformer [33] and PARENet [47] utilize their official KITTI pre-trained weights with uniformly downsampled inputs exhibit suboptimal performance, achieving only 38.89% and 71.30% success rates, respectively, under the 1:10 ratio. This progressive performance degradation is caused by the scale unbalance increasing. This limitation stems from their inability to handle the extreme point density variation and heterogeneous geometric information in unbalanced scenarios, a fundamental challenge that current learning-based architectures struggle to address. Our method outperforms all competing methods by achieving the highest registration success rate while simultaneously maintaining significantly superior computational efficiency compared with other high-performance methods. The comparative visualization of registration results for all methods is presented in Figure 3, featuring three representative scenarios from the KITTI-UPP dataset.

To further investigate performance boundaries under extreme unbalanced conditions, we conducted an additional experiment with an unprecedented unbalanced ratio of 1:20, which registers a 150-frame query point cloud against a 3000-frame reference point cloud. The scenario presents substantial challenges due to the massive scale disparity and significantly amplified outlier ratios. We selectively compared our method with the most competitive methods, including Fast-MAC [43], MAC [41], SC2-PCR [42], and Teaser++ [45], which had demonstrated superior performance in prior experiments. As evidenced in Table 3, our method achieves the highest registration success rate of 94.44% with exceptional computational efficiency of 0.11 s. Our method demonstrates a 2.77% point improvement in registration accuracy over SC2-PCR [42], which achieved 91.67% accuracy with 0.16 s computation time. Similarly, our method outperforms Teaser++ [45], MAC [41], and Fast-MAC [43] by 8.33%, 11.11%, and 14.81% points in registration success rate, respectively, while maintaining substantially lower computational requirements. These compelling results conclusively demonstrate notable competitiveness and exceptional robustness even in the ultra-extreme scenario of our method, underscoring the effectiveness of our curvature-aware method in handling severe scale disparities while ensuring computational efficiency.

### 4.3. Results on TIESY Dataset

#### 4.3.1. Evaluation on One-to-Many Correspondences

Building upon our evaluation framework, this study conducts an evaluation of one-to-many correspondence generation using ISS [22] keypoints on the real-world TIESY Dataset with ratios ranging from 1:4 to 1:6. The assessment examines two distinct registration scenarios: *(1) Small-to-Large*: registration from the partial scans to the whole; *(2) Large-to-Small*: registration from the whole scans to the partial;

In the experiments, the parameter *N* is varied across the following discrete values:

**Small-to-Large**: N∈{1,2,4,8,12,16,22,26};

**Large-to-Small**: N∈{1,2,3,4}.

Six point cloud registration methods, including Fast-MAC [43], MAC [41], SC2-PCR [42], Teaser++ [45], FGR [46] and RANSAC [28] are rigorously evaluated under both configurations. Comprehensive performance results, including registration success rates and error metrics, are provided in Table 4.

While the RR of Fast-MAC [43], MAC [41], and Teaser++ [45] exhibited a positive correlation with increasing *N*, though with commensurate computational time escalation—for instance, in the partial-to-whole registration scenario, as *N* increased from 1 to 12, the RR improved significantly from 68.42% to 86.32%. Conversely, FGR [46] and SC2-PCR [42] maintained stable performance across all *N* configurations, while RANSAC [28] demonstrated progressive performance degradation as correspondence quantities expanded. In contrast, our method consistently achieves the highest registration success rate with the fastest computational speed, highlighting its superior efficiency and robustness under varying correspondence densities.

The following conclusions can be drawn: As *N* increases, the inlier count of correspondences increases, while the inlier ratio decreases. In practical registration, this effectively improves the registration success rate, but this comes at the cost of significantly increased computation time.

#### 4.3.2. Robust Experiments

We conducted extensive experiments on the real-world TIESY dataset under both noiseless and noisy conditions, evaluating three unbalanced registration scenarios. Our evaluation framework is built on the CURV-based keypoint detection. For comprehensive comparison, we evaluate both traditional methods: Fast-MAC [43], MAC [41], SC2-PCR [42], Teaser++ [45], FGR [46], and RANSAC [28], and deep learning-based methods including GeoTransformer [33] and PARENet [47], and the results are represented in Table 5. Notably, the deep learning-based methods are evaluated using their official KITTI pre-trained weights, as TIESY is also an outdoor dataset. However, as shown in Table 5, these learning-based methods demonstrate significantly inferior performance, achieving only 39.80% and 33.67% registration success rates, respectively, substantially lower than traditional high-performance methods. The performance gap can be attributed to two primary factors: The TIESY dataset features substantial environmental complexity, comprising a wide range of real-world scenarios from buildings to vegetation. This diversity reveals the limited generalization capacity of deep learning-based methods when faced with challenging and varied conditions.

We quantitatively evaluate the noise robustness of our method by testing with different levels of Gaussian noise. The results presented in Table 6 demonstrate that our method maintains competitive performance across various noise conditions while consistently achieving low computational time. As indicated, while our method maintains strong performance at lower noise levels with a registration rate of 93.68% at noise level 0.01, a noticeable degradation occurs at higher noise intensities where the registration rate drops to 88.42% at noise level 0.03. This performance decline can be attributed to several factors inherent in the local geometric verification process. Firstly, the curvature-based keypoint detection, while robust under moderate noise, becomes less stable when significant Gaussian noise distorts local surface geometries, leading to inconsistent keypoint repeatability. Secondly, the FPFH descriptors, though efficient, are sensitive to neighborhood perturbations caused by noise, which amplifies feature mismatching in the one-to-many correspondence generation stage. Finally, the local structure similarity matching, which relies on precise distance and angular relationships, suffers from threshold misalignment when noise exceeds the adaptive tolerance of the geometric verification module.

### 4.4. Ablation Study

#### 4.4.1. Keypoint Detection Comparison

We conduct experimental analyses on the KITTI-UPP and real-world TIESY datasets, specifically evaluating the performance of the keypoint detection methods proposed in Section 3—namely ISS [22], H3D [23], and curvature-based detection—combined with FPFH for generating various correspondences. The results of our tests on the KITTI-UPP dataset are summarized in Table 7, where the values of N for the small-to-large and large-to-small sequences are set to {1, 2, 4, 8, 12, 24} and {1, 2, 3, 4, 5, 6}, respectively. It can be observed that the curvature-based method, when combined with FPFH, significantly outperforms the other two methods in terms of correspondence quality and overall registration accuracy. As presented in Table 8, the inlier statistics of curvature-based feature matching on the real-world TIESY dataset are summarized. For this dataset, the values of N for the small-to-large and large-to-small sequences are set to {1, 2, 4, 8, 12, 16, 22, 26} and {1, 2, 3, 4}, respectively. Although the inlier ratio naturally decreases as the value of N increases, the absolute number of inliers demonstrates a consistent growing trend. This observation indicates that the feature correspondences derived from CURV keypoint detection exhibit high quality, enabling the algorithm to effectively distill a substantial number of correct correspondences from a large pool of candidate matches. These reliable correspondences form a solid foundation for subsequent high-precision registration tasks.

#### 4.4.2. Parameter Sensitivity Analysis

We conducted an ablation study on the KITTI-UPP(1:4) dataset to evaluate the robustness of our method under different parameter settings. With fixed thresholds (distance difference d<0.1 m, angular difference $\theta <5^{\circ }$, and matching score =1 in Section 4, we systematically varied the number of neighboring points *k* around each target point. As shown in Table 9, with our parameter N set to 24, the method demonstrates strong robustness across different *k* values, maintaining high registration rates above 95% while keeping rotation errors below 0.007 rad in all cases. The consistent performance across parameter variations confirms the stability of our method.

To validate our core innovative components and assess the generalization capability of our method, we conduct ablation studies on KITTI-UPP(1:4) with parameter k=4. We examine individual and combined contributions of angle and distance constraints across FPFH and PARENet descriptors using 1-to-24 correspondence pairs. As shown in Table 10, the combined constraints achieve optimal performance for both descriptors. FPFH with combined constraints reaches 100.00% RR, significantly outperforming angle-only (92.59%) and distance-only (25.00%) configurations. PARENet similarly benefits from combination (93.52%) versus angle-only (77.78%) and distance-only (85.19%). The complementary nature of constraints is evident from their varying effectiveness across descriptors. These results demonstrate that our method, through its combined use of angle and distance, is not only effective across different descriptors but also crucial for delivering superior registration accuracy in unbalanced point clouds.

## 5. Conclusions

We proposed a curvature-aware outlier rejection method for unbalanced point cloud registration. Our method employs a one-to-many correspondence strategy to increase inlier counts using geometric redundancy while preserving structural invariance. Based on local geometric consistency, we developed a robust outlier removal mechanism for dense correspondence sets. Experiments on synthetic KITTI-UPP and real-world TIESY datasets demonstrated that the proposed method achieved inherent resilience to partial overlaps through probabilistic correspondence expansion and improved registration stability via geometrically verified candidate pooling. The results confirmed that our correspondence generation strategy successfully balanced match quantity and precision in unbalanced registration. However, the proposed method exhibited relatively weak robustness in keypoint detection due to its reliance on curvature-based features. The future work will focus on enhancing keypoint stability, algorithmic acceleration for ultra-dense scenarios, integrating multi-scale or global features to handle large-scale inconsistencies, reducing sensitivity to parameters via adaptive- or learning-based optimization, and improving computational efficiency through hierarchical pruning and optimized feature extraction.

## Figures and Tables

**Table 1:** Evaluation of one-to-many correspondences on KITTI-UPP benchmark for both scenarios. **Bold** indicates the best result and underline indicates the second-best. `/` indicates cases where RR is 0%, making RE, TE, and Time inapplicable. `Ours*` employs ISS [22] in place of the CURV-based keypoint detection.

(a) Small-to-Large Scenario (Partial-to-Whole)																
Method	N = 1				N = 4				N = 6				N = 8			
	RR (%)	RE (°)	TE (cm)	Time (s)	RR (%)	RE (°)	TE (cm)	Time (s)	RR (%)	RE (°)	TE (cm)	Time (s)	RR (%)	RE (°)	TE (cm)	Time (s)
RANSAC [28]	7.41	0.09	15.12	**0.01**	2.78	0.08	18.31	**0.01**	0.00	/	/	/	0.00	/	/	/
FGR [46]	39.81	2.25	42.35	0.34	41.67	1.81	45.56	1.28	41.67	2.24	47.58	2.13	42.59	2.39	55.21	2.88
Teaser++ [45]	57.41	9.03	36.21	0.07	**82.41**	0.02	9.56	0.32	87.04	**0.01**	4.11	0.87	92.59	0.03	5.63	1.49
SC2-PCR [42]	57.41	0.08	25.15	**0.01**	70.37	0.02	6.21	0.07	66.67	0.17	51.21	0.11	65.74	0.02	2.15	0.13
MAC [41]	52.78	**0.01**	44.12	0.10	**82.41**	**0.01**	14.52	2.30	87.96	0.02	13.25	5.10	94.44	**0.01**	25.69	10.65
Fast-MAC [43]	51.85	**0.01**	45.21	**0.01**	79.63	**0.01**	16.32	0.11	85.19	0.02	17.35	1.29	89.81	**0.01**	27.95	3.48
Ours*	35.19	0.11	34.44	0.10	62.04	0.06	19.12	0.10	78.70	0.02	0.82	**0.10**	88.88	0.02	4.82	**0.10**
Ours	**70.37**	**0.01**	**2.85**	0.09	**95.37**	**0.01**	**0.25**	0.10	**99.07**	**0.01**	**0.13**	**0.10**	**99.07**	**0.01**	**0.17**	**0.10**
**Method**	**N = 12**				**N = 16**				**N = 20**				**N = 24**			
	**RR (%)**	**RE (°)**	**TE (cm)**	**Time (s)**	**RR (%)**	**RE (°)**	**TE (cm)**	**Time (s)**	**RR (%)**	**RE (°)**	**TE (cm)**	**Time (s)**	**RR (%)**	**RE (°)**	**TE (cm)**	**Time (s)**
RANSAC [28]	0.00	/	/	/	0.00	/	/	/	0.00	/	/	/	0.00	/	/	/
FGR [46]	37.96	2.23	54.47	4.15	30.81	2.48	40.60	5.08	34.26	2.08	41.66	6.30	37.96	2.64	56.32	7.60
Teaser++ [45]	96.30	0.02	5.01	2.72	98.15	**0.01**	3.69	5.09	**100.00**	**0.01**	4.52	8.77	61.11	**0.01**	3.24	15.83
SC2-PCR [42]	51.85	0.02	3.35	0.14	50.00	0.11	53.31	0.16	43.37	0.13	51.63	0.18	43.52	0.02	6.87	0.19
MAC [41]	97.22	**0.01**	18.56	44.26	87.04	**0.01**	31.32	68.25	51.85	**0.01**	31.85	79.05	26.85	0.02	30.21	95.14
Fast-MAC [43]	89.81	**0.01**	20.21	4.20	63.89	**0.01**	32.25	17.79	38.89	0.02	36.21	5.37	19.44	0.02	36.68	7.55
Ours*	97.22	**0.01**	3.58	0.11	97.22	**0.01**	4.02	**0.11**	98.15	0.05	6.52	**0.11**	99.07	**0.01**	3.54	**0.11**
Ours	**99.07**	**0.01**	**0.14**	**0.10**	**100.00**	**0.01**	**0.08**	**0.11**	**100.00**	**0.01**	**0.09**	**0.11**	**100.00**	**0.01**	**0.06**	**0.11**
**(b) Large-to-Small Scenario (Whole-to-Partial)**																
**Method**	**N = 1**				**N = 2**				**N = 4**				**N = 6**			
	**RR (%)**	**RE (°)**	**TE (cm)**	**Time (s)**	**RR (%)**	**RE (°)**	**TE (cm)**	**Time (s)**	**RR (%)**	**RE (°)**	**TE (cm)**	**Time (s)**	**RR (%)**	**RE (°)**	**TE (cm)**	**Time (s)**
RANSAC [28]	0.93	**0.02**	10.36	**0.01**	0.93	0.09	15.36	**0.02**	0.00	/	/	/	0.00	/	/	/
FGR [46]	37.04	2.99	44.63	1.18	37.96	2.85	48.89	2.35	25.93	2.24	48.62	4.74	36.11	3.15	42.31	5.17
Teaser++ [45]	85.19	0.09	19.68	0.26	91.67	0.03	6.35	1.03	69.44	0.02	5.21	3.06	71.30	0.04	9.56	7.17
SC2-PCR [42]	65.74	0.10	27.58	0.06	59.26	0.05	6.84	0.12	44.44	0.12	25.01	0.15	48.15	0.04	10.25	0.18
MAC [41]	79.63	**0.02**	4.12	2.00	91.67	**0.01**	5.78	8.92	69.44	0.01	7.71	32.53	72.22	0.01	8.25	97.05
Fast-MAC [43]	75.00	**0.02**	4.69	0.10	85.19	**0.01**	17.86	6.94	64.81	0.02	8.96	4.39	62.96	0.01	8.63	8.47
Ours*	47.22	0.04	9.01	0.10	83.33	**0.01**	7.03	0.10	78.70	0.03	5.86	**0.11**	87.96	0.05	7.52	**0.11**
Ours	**98.15**	**0.02**	**1.42**	0.10	**100.00**	**0.01**	**0.78**	0.10	**100.00**	**0.00**	**0.82**	**0.11**	**100.00**	**0.00**	**0.68**	**0.11**

**Table 2:** Registration results on KITTI-UPP dataset with different keypoint detection modules. **Bold** indicates the best result and underline indicates the second-best.

	Correspondence	Method	1:10 Ratio		1:4 Ratio		1:1 Ratio	
			RR (%)	Time (s)	RR (%)	Time (s)	RR (%)	Time (s)
(i) Deep learning	-	GeoTransformer [33]	38.89	4.65	62.04	2.29	94.44	0.62
		PARENet [47]	71.30	9.01	87.04	4.05	**100.00**	0.78
(ii) Traditional	ISS+FPFH [22]	RANSAC [28]	0.00	**0.01**	0.00	**0.01**	63.89	**0.01**
		FGR [46]	43.52	1.19	37.96	4.15	94.44	1.06
		Teaser++ [45]	81.48	0.45	96.30	2.72	95.37	0.90
		SC2-PCR [42]	74.77	0.11	51.85	0.14	96.30	0.04
		MAC [41]	88.66	9.90	97.22	44.26	94.44	2.02
		Fast-MAC [43]	84.11	0.19	89.81	4.20	94.44	1.02
	H3D+FPFH [23]	RANSAC [28]	0.00	**0.01**	0.00	**0.01**	47.22	**0.01**
		FGR [46]	50.00	5.60	64.81	2.87	**100.00**	0.62
		Teaser++ [45]	83.33	1.98	89.81	1.16	**100.00**	2.33
		SC2-PCR [42]	70.37	0.39	83.33	0.26	97.22	0.02
		MAC [41]	93.51	26.52	94.44	11.47	**100.00**	3.44
		Fast-MAC [43]	92.94	4.18	91.67	3.07	**100.00**	1.58
	CURV+FPFH	RANSAC [28]	0.00	**0.01**	8.33	**0.01**	95.37	**0.01**
		FGR [46]	84.26	5.10	87.96	3.48	**100.00**	0.99
		Teaser++ [45]	90.74	7.49	93.52	9.61	**100.00**	0.20
		SC2-PCR [42]	94.44	0.14	**99.07**	0.11	**100.00**	0.30
		MAC [41]	89.81	24.96	87.04	15.78	**100.00**	1.09
		Fast-MAC [43]	87.96	6.46	87.04	2.78	**100.00**	0.15
		Ours	**95.37**	0.10	**99.07**	0.10	**100.00**	0.11

**Table 3:** Registration results on KITTI-UPP dataset under 1:20 unbalanced ratio with best-performing methods. **Bold** indicates the best result and underline indicates the second-best. (150-frame query vs. 3000-frame reference).

Method	RR (%)	Time (s)
TEASER++ [45]	86.11	3.38
SC2-PCR [42]	91.67	0.16
MAC [41]	83.33	25.11
Fast-MAC [43]	79.63	4.15
Ours	**94.44**	**0.11**

**Table 4:** Evaluation of one-to-many correspondences on real-world TIESY benchmark for both scenarios. **Bold** indicates the best result and underline indicates the second-best. `-` indicates untested cases due to memory overflow or prohibitive computation time.

(a) Small-to-Large Scenario (Partial-to-Whole)																
Method	N = 1				N = 2				N = 4				N = 8			
	RR (%)	RE (°)	TE (cm)	Time (s)	RR (%)	RE (°)	TE (cm)	Time (s)	RR (%)	RE (°)	TE (cm)	Time (s)	RR (%)	RE (°)	TE (cm)	Time (s)
RANSAC [28]	37.89	0.06	21.25	**0.01**	30.53	0.10	27.54	**0.01**	27.37	0.18	45.51	**0.01**	16.84	0.09	29.63	**0.02**
FGR [46]	47.37	0.72	45.85	0.53	46.32	0.63	41.44	0.75	42.11	0.75	46.35	0.67	41.05	0.88	41.01	2.26
Teaser++ [45]	69.47	**0.01**	1.36	0.03	75.79	**0.00**	0.98	0.08	84.21	**0.00**	1.55	0.26	84.21	0.02	6.36	1.01
SC2-PCR [42]	68.42	**0.01**	0.68	0.82	69.47	0.02	0.87	0.23	75.79	0.02	0.95	0.25	75.79	0.02	**0.64**	0.30
MAC [41]	68.42	**0.01**	5.21	0.46	72.63	0.01	8.62	1.35	83.16	0.01	18.54	2.59	85.26	**0.01**	18.75	11.71
Fast-MAC [43]	69.15	**0.01**	4.87	0.02	71.58	0.01	5.34	0.06	78.95	0.01	5.63	0.23	85.26	**0.01**	10.98	9.47
Ours*	63.82	**0.01**	0.89	0.09	67.02	0.01	1.02	0.09	74.47	**0.00**	0.45	0.10	84.21	0.02	0.82	0.10
Ours	**72.34**	**0.01**	**0.47**	0.09	**79.79**	0.01	**0.65**	0.10	**86.17**	0.01	**0.82**	0.10	**92.55**	0.02	0.77	0.10
**Method**	**N=12**				**N=16**				**N=22**				**N=26**			
	**RR (%)**	**RE (°)**	**TE (cm)**	**Time (s)**	**RR (%)**	**RE (°)**	**TE (cm)**	**Time (s)**	**RR (%)**	**RE (°)**	**TE (cm)**	**Time (s)**	**RR (%)**	**RE (°)**	**TE (cm)**	**Time (s)**
RANSAC [28]	13.68	0.41	48.24	**0.02**	10.53	0.62	41.36	**0.02**	9.47	1.19	48.69	**0.03**	6.32	0.66	48.56	**0.03**
FGR [46]	38.95	1.46	40.08	3.30	33.68	1.09	39.62	4.36	30.53	1.18	41.14	5.94	30.53	1.24	40.05	6.98
Teaser++ [45]	86.32	**0.00**	1.08	2.32	–	–	–	–	–	–	–	–	–	–	–	–
SC2-PCR [42]	69.47	0.02	0.85	0.32	67.37	**0.01**	0.92	0.34	66.32	0.04	6.01	0.36	68.42	0.04	8.08	0.38
MAC [41]	86.32	0.01	16.52	29.84	–	–	–	–	–	–	–	–	–	–	–	–
Fast-MAC [43]	86.32	0.01	17.48	54.82	–	–	–	–	–	–	–	–	–	–	–	–
Ours*	86.32	0.01	0.95	0.10	87.23	**0.01**	**0.68**	0.10	89.36	**0.01**	1.20	0.10	90.43	**0.01**	1.04	0.10
Ours	**92.55**	0.01	**0.65**	0.10	**94.68**	0.06	**0.68**	0.10	**96.81**	0.02	**0.59**	0.10	**96.81**	**0.01**	**0.49**	0.10
**(b) Large-to-Small Scenario (Whole-to-Partial)**																
**Method**	**N = 1**				**N = 2**				**N = 3**				**N = 4**			
	**RR (%)**	**RE (°)**	**TE (cm)**	**Time (s)**	**RR (%)**	**RE (°)**	**TE (cm)**	**Time (s)**	**RR (%)**	**RE (°)**	**TE (cm)**	**Time (s)**	**RR (%)**	**RE (°)**	**TE (cm)**	**Time (s)**
RANSAC [28]	12.63	0.57	14.21	**0.02**	10.53	1.16	19.58	**0.03**	5.26	1.15	22.35	**0.03**	5.26	2.84	34.52	**0.03**
FGR [46]	35.79	0.90	52.01	1.92	33.68	1.18	54.45	3.72	34.74	1.17	49.86	5.56	31.58	1.13	48.78	7.27
Teaser++ [45]	81.05	**0.01**	0.87	0.97	–	–	–	–	–	–	–	–	–	–	–	–
SC2-PCR [42]	72.63	0.22	42.24	0.17	56.84	0.11	9.87	0.33	54.74	0.07	20.06	0.36	45.26	0.09	27.14	0.39
MAC [41]	81.05	**0.01**	0.45	12.22	91.49	**0.01**	0.86	26.23	–	–	–	–	–	–	–	–
Fast-MAC [43]	78.95	**0.01**	**0.58**	16.06	90.53	0.02	0.97	82.60	–	–	–	–	–	–	–	–
Ours*	73.40	**0.01**	1.29	0.10	85.11	0.02	1.05	0.11	88.29	**0.01**	3.58	0.10	92.55	**0.01**	2.25	0.10
Ours	**90.43**	**0.01**	1.01	0.10	**92.55**	**0.01**	**0.85**	0.10	**93.62**	**0.01**	**0.82**	0.10	**93.62**	**0.01**	**0.55**	0.10

**Table 5:** Registration results of various methods on real-world TIESY dataset. **Bold** indicates the best result and underline indicates the second-best.

	Method	RR (%)	Time (s)
(i) Deep Learning	GeoTransformer [33]	39.80	8.24
	PARENet [47]	33.67	12.49
(ii) Traditional	RANSAC [28]	8.42	**0.02**
	FGR [46]	72.63	1.63
	Teaser++ [45]	**95.79**	0.58
	SC2-PCR [42]	93.68	0.14
	MAC [41]	**95.79**	7.00
	Fast-MAC [43]	93.68	0.26
	Ours	**95.79**	0.11

**Table 6:** Registration results of our method on real-world TIESY dataset under various noise settings.

Noise Level	RR (%)	Time (s)
0.01	93.68	0.11
0.02	95.79	0.11
0.03	88.42	0.11

**Table 7:** The comparison of various keypoint detection models on KITTI-UPP datasets.

Keypoint	Registration	Metric	*N*					
			1	2	4	8	12	24
CURV	Small-to-Large	Correspondence Count	1574.33	3148.66	6297.32	12,594.64	18,891.96	37,783.92
		Inlier Ratio (%)	10.02	7.21	4.29	2.50	1.83	1.09
		Inlier Count	161.13	230.87	274.63	320.36	348.62	389.35
	Large-to-Small	Correspondence Count	5169.46	10,338.92	15,508.38	20,677.84	25,847.30	31,016.76
		Inlier Ratio (%)	3.83	2.78	2.09	1.70	1.44	1.26
		Inlier Count	191.80	278.13	311.79	337.57	357.83	375.38
H3D [23]	Small-to-Large	Correspondence Count	1174.05	2348.10	4696.20	9392.40	14,088.60	28,177.20
		Inlier Ratio (%)	2.20	1.49	1.02	0.68	0.55	0.37
		Inlier Count	25.09	33.44	44.35	57.15	65.74	84.17
	Large-to-Small	Correspondence Count	4810.85	9621.70	14,432.55	19,243.40	24,054.25	28,865.10
		Inlier Ratio (%)	0.85	0.60	0.49	0.42	0.37	0.33
		Inlier Count	36.57	50.03	59.86	67.56	74.19	77.48
ISS [22]	Small-to-Large	Correspondence Count	1715.88	3431.76	6863.52	13,727.04	20,590.56	41,181.12
		Inlier Ratio (%)	1.20	0.86	0.59	0.39	0.31	0.21
		Inlier Count	21.56	30.86	41.75	54.93	65.29	88.81
	Large-to-Small	Correspondence Count	5951.96	11,903.92	17,855.88	23,807.84	29,759.80	35,711.76
		Inlier Ratio (%)	0.56	0.40	0.33	0.28	0.25	0.23
		Inlier Count	32.72	47.23	57.52	65.93	73.24	80.48

**Table 8:** Inlier statistics of our method on real-world TIESY dataset. `-` indicates no data.

Registration Mode	Metric	*N*							
		1	2	4	8	12	16	22	26
Small-to-Large	Correspondence Count	1540.93	3081.86	6163.72	12,327.44	18,491.16	24,654.88	33,900.46	40,064.18
	Inlier Ratio (%)	10.15	6.37	3.99	2.51	1.93	1.60	1.30	1.16
	Inlier Count	141.23	176.86	219.87	273.60	313.26	344.47	383.58	405.41
Large-to-Small	Correspondence Count	10,641.47	21,262.94	31,924.41	42,565.88	–	–	–	–
	Inlier Ratio (%)	2.15	1.44	1.14	0.96	–	–	–	–
	Inlier Count	179.47	234.11	272.47	302.97	–	–	–	–

**Table 9:** Analysis experiment of our method with different *k* values on the KITTI-UPP(1:4) dataset.

*k*	RR	RE	TE	Time
3	95.37%	0.0070	0.037	0.1491
4	100.00%	0.0002	2.563	0.1488
5	100.00%	0.0002	1.763	0.1487
6	99.07%	0.0002	1.130	0.1487
7	99.07%	0.0002	9.701	0.1487
8	98.15%	0.0001	4.670	0.1488

**Table 10:** Ablation study on geometric verification components with different descriptors on KITTI-UPP(1:4) dataset. **Bold** indicates the best result and underline indicates the second-best. (AC: Angle Constraint; DC: Distance Constraint).

Descriptor	AC	DC	RR (%)	RE (°)	TE (cm)	Time (s)
FPFH [24]	✓		92.59	0.0008	**0.002**	**0.1144**
		✓	25.00	**0.0002**	6.637	0.1153
	✓	✓	**100.00**	**0.0002**	2.563	0.1488
PARENet [47]	✓		77.78	0.0336	0.156	0.0967
		✓	85.19	0.0030	**0.008**	**0.0949**
	✓	✓	**93.52**	**0.0002**	9.032	0.0975

## Data Availability

The data used to support the findings of this study are included in the article. The source code and dataset will be available at https://github.com/SHIYI-hu/Curvature-Aware-Point-Pair-Signatures (accessed on 6 October 2025).
